# Edaravone prevents high glucose-induced injury in retinal Müller cells through thioredoxin1 and the PGC-1α/NRF1/TFAM pathway

**DOI:** 10.1080/13880209.2021.1972123

**Published:** 2021-09-10

**Authors:** Juanping Yin, Xinke Chen

**Affiliations:** a Department of Ophthalmology, The Fourth Hospital of Changsha, Changsha Hospital of Hunan Normal University, Changsha, China; b Department of Ophthalmology, Children’s Hospital of Chongqing Medical University, National Clinical Research Center for Child Health and Disorders, Ministry of Education Key Laboratory of Child Development and Disorders, Chongqing Key Laboratory of Pediatrics, Chongqing, China

**Keywords:** Diabetes, retina, ROS, antioxidant, apoptosis, mitochondrial membrane potential, oxidative products, antioxidative enzymes

## Abstract

**Context:**

Oxidative injury in a high-glucose (HG) environment may be a mechanism of diabetic retinopathy (DR) and edaravone can protect retinal ganglion cells by scavenging ROS.

**Objective:**

To explore the effect of edaravone on HG-induced injury.

**Materials and methods:**

First, Müller cells were cultured by different concentrations of glucose for different durations to obtain a suitable culture concentrations and duration. Müller cells were then divided into Control, HG + Vehicle, HG + Eda-5 μM, HG + Eda-10 μM, HG + Eda-20 μM, and HG + Eda-40 μM groups. Cells were cultured by 20 mM glucose and different concentrations of edaravone for 72 h.

**Results:**

The IC_50_ of glucose at 12–72 h is 489.3, 103.5, 27.92 and 20.71 mM, respectively. When Müller cells were cultured in 20 mM glucose for 72 h, the cell viability was 52.3%. Edaravone significantly increased cell viability compared to Vehicle (68.4% vs 53.3%; 78.6% vs 53.3%). The EC_50_ of edaravone is 34.38 μM. HG induced high apoptosis rate (25.5%), while edaravone (20 and 40 μM) reduced it to 12.5% and 6.89%. HG increased the DCF fluorescence signal (189% of Control) and decreased the mitochondrial membrane potential by 57%. Edaravone significantly decreased the DCF fluorescence signal (144% and 132% of Control) and recovered the mitochondrial membrane potential to 68% and 89% of Control. Furthermore, HG decreased the expression of TRX1, PGC-1α, NRF1 and TFAM, which were restored by edaravone.

**Discussion and conclusion:**

These findings provide a new potential approach for the treatment of DR and indicated new molecular targets in the prevention of DR.

## Introduction

With the development of social economy, diabetes has become one of the important diseases threatening human health. It is estimated that, by 2040, there may be >600 million patients with diabetes worldwide (Arroba et al. 2016). As an important ocular complication of diabetes, diabetic retinopathy (DR) has an overall incidence rate of 35% in patients with DM, and it has become an important disease, causing blindness in adults (Huang et al. 2010). However, there is still a lack of clinically effective drugs and treatments for retinopathy. Therefore, studying the pathogenesis, prevention, and treatment of DR has important clinical significance.

In recent years, it was proposed that retinal neurodegeneration is the earliest pathological change in retinopathy (Simó et al. 2012). The indicators of neurodegeneration of the retina are glial proliferation of retinal Müller cells, loss of ganglion cells, thinning of the nuclear layer, and apoptosis of neurons and photoreceptor cells (Chaum 2003; Kuhrt et al. 2008). Müller cells account for the majority of retinal glial cells, and play an essential role in the maintenance of the retinal function. The radial protrusions of Müller cells provide structural and functional support for the exchange of nutrients and metabolic waste between neurons and blood vessels, thus affecting the integrity of the blood-retinal barrier and maintaining neuronal activity. Previous studies suggested that Müller cells may function as retinal stem cells under special transformation conditions (Verma et al. 2009; Boynton et al. 2015). In addition, Müller cells can secrete a variety of cytokines to provide nutritional support for retinal neurons and enable them to perform normal physiological functions. Under a long-term hyperglycaemic environment, the number of glial cells, ion channels and receptor expression become affected, which is manifested as abnormal glutamate metabolism and increased glial cell apoptosis (Verma et al. 2009; Boynton et al. 2015).

In recent years, multiple studies have shown that excessive generation of ROS is a direct result of hyperglycaemia and the main factor leading to the microvascular complications of diabetes (Rösen et al. 2001; Brownlee 2005; Rolo and Palmeira 2006). Various studies have proposed that excessive mitochondrial ROS generation in a high-glucose environment and cellular oxidative stress damage may be the common mechanism of diabetes complications (Brownlee 2005; Rolo and Palmeira 2006). It has been shown that abnormalities in mitochondrial structure and function can initiate the apoptosis pathway and cause various complications of diabetes such as peripheral neuropathy, cardiomyopathy and renal impairment (Cai et al. 2002; Russell et al. 2002; Verzola et al. 2002). The mitochondrial membrane potential refers to the difference in transmembrane potential caused by different ion concentrations on both sides of the mitochondrial membrane. It is a necessary condition for maintaining the normal biological function of mitochondria, thus reflecting the integrity of mitochondrial function, and is a sensitive indicator for evaluating the normality of mitochondrial function.

Edaravone is a neuroprotective drug that was first approved by Japan for the treatment of acute cerebral ischaemia and infarction disease. It is a powerful antioxidant that has a strong free radical scavenging ability and can protect tissues from oxidative stress (Yoshida et al. 2006). Several studies have shown that edaravone can protect retinal ganglion cells in rat models of retinal detachment and mouse models of light-induced damage by scavenging ROS (Hironaka et al. 2011; Shimazaki et al. 2011; Xu et al. 2017). However, there are no studies on the protective effect of edaravone on animal models of DR. Thioredoxin (TRX) was first identified by Laurent et al. (1964) as an enzyme necessary for DNA synthesis. It was later confirmed to be widely distributed in mammalian tissues. TRX plays an important role in numerous biological processes, including antioxidation, anti-apoptosis, immune response and virus infection (Lu and Holmgren 2014). TRX, TRX reductase (TRXR) and NADPH form the TRX system (Lu and Holmgren 2014), which works with glutathione (GSH), peroxiredoxin and other molecules to maintain the redox balance in the cell, protecting cells from oxidative stress (Förstermann 2008). It can also regulate the expression of a variety of genes and induce the production of multiple enzymes with biological activity (Dunn et al. 2010). However, the association between edaravone and TRX in high-glucose induced cell injury has not been studied yet.

Based on previous studies, we raised a hypothesis that edaravone may protect retinal Müller cells against high-glucose induced injury. Therefore, the present study cultured rat retinal Müller cells in a high-glucose environment *in vitro* and used them as an *in vitro* model of retinopathy to explore the protective effect of edaravone on high-glucose induced injury in retinal Müller cells in order to provide new potential treatments for retinopathy. The involvement of TRX and the PGC-1α/NRF1/TFAM pathway was investigated to clarify the mechanism.

## Materials and methods

### Primary Müller cell cultures

Primary Müller cells were prepared from eyes of ten postnatal 10–21-day-old Sprague-Dawley rats (Das et al. 2006). The eyeballs were removed and washed in alcohol, and then immersed in D-Hank’s solution. After being rinsed three times, the retina were separated and placed into a centrifuge tube. Next, 0.25% trypsin was added for digestion during 10 min. The samples were then filtered with a 150-mesh screen, and centrifuged at 12,000 ×*g* at room temperature for 5 min. The cell pellet was then collected and resuspend in DMEM containing 20% ​​fetal bovine serum (Beyotime, Shanghai, China). Next, the cells were dispersed into a single cell suspension, inoculated in a 50 mL culture flask at a cell density of 3 × 10^5^ cells/mL, and incubated in a 37 °C in a 5% CO_2_ incubator. The medium was changed after the cells adhered to the wall. The study was approved by the Ethics Committee of Chongqing Medical University (No. 2018-5789).

### Identification of Müller cells

Identification of Müller cells was performed after passaging the cells to the third generation. A cover glass was placed in a Petri dish. Next, the cells were digested and seeded on the cover glass. When the cells grew close to confluence, they were removed, washed with D-Hank’s solution, fixed with 4% paraformaldehyde (20 min) and treated with 0.1% Triton (15 min). The endogenous peroxidase was blocked by incubating the cells with 0.3% H_2_O_2_ for 20 min. After being blocked with normal goat serum (Beyotime, Shanghai, China) for 20 min, mouse anti-glial fibrillary acidic protein (GFAP, 1:200; Sigma-Aldrich, USA) or rabbit anti-glutamine synthetase (GS, 1:1,000; Sigma-Aldrich, USA) was added, while PBS (0.01 mol/L, pH 7.2) was added to the control group. The samples were incubated overnight at 4 °C and eluted with PBS. A goat anti-rat IgG-HRP was used as the secondary antibody (No. sc-2005, 1:3000; Santa Cruz Biotechnology, Santa Cruz, CA, USA, 1 h under room temperature). Upon elution with PBS, Hoechst was added to stain the nucleus. Cells were observed under a fluorescence inverted phase contrast microscope. The immunofluorescence staining showed that the majority of cells were GFAP- and GS-positive, indicating the purity of cells. The cell bodies and protrusions emitted red fluorescence, while the nucleus was Hoechst-positive with blue fluorescence, indicating that these cells were Müller cells (>90% of cells were identified as Müller cells).

### High glucose and edaravone treatment

To obtain a suitable incubation concentration and duration of glucose, primary Müller cells were cultured in DMEM containing different concentrations of glucose (5, 10, 20, and 40 mM) for different durations (12, 24, 48, and 72 h). DMEM was supplemented with mannitol to balance the osmosis pressure. Mannitol was used as vehicle, which was the same for all the groups. The medium was changed daily. Cell viability was measured by the CCK-8 method.

For edaravone treatment, edaravone was added to DMEM containing 20 mM glucose to obtain different concentrations (5, 10, 20, and 40 μM). Müller cells were cultured in the above DMEM for 72 h. The medium was changed daily. In the vehicle group, the same volume of saline was added.

### Cell viability assay

Cell viability was measured by a CCK-8 kit (Beyotime, Shanghai, China). Briefly, Müller cells were seeded in a 96-well plate (5000 cells/well, 100 μL). DMEM containing different concentrations of glucose or edaravone was added to the wells. The medium was changed daily. After the incubation was completed, 10 μL CCK-8 working solution was added into each well and kept for 2 h. Finally, the absorbance at 450 nm was measured to calculate the cell viability.

### Analysis of cell apoptosis

The analysis of cell apoptosis was performed with a commercial apoptosis detection kit (Beyotime, Shanghai, China). After culturing the Müller cells in DMEM containing high glucose and edaravone for 72 h, cells were subjected to dual staining with FITC-conjugated Annexin V and PI (Beyotime, Shanghai, China) according to the manufacturer’s instructions. The apoptosis rate was then detected with a flow cytometer (Thermo Fisher Scientific, Inc, MA, USA).

### Western blot analysis

After culturing the Müller cells in DMEM containing high glucose and edaravone for 72 h, the cells were collected and lysed with cell lysis buffer (Beyotime, Shanghai, China). The proteins were collected and separated by SDS-PAGE. Electrophoresis was conducted at 60 V for 2 h and 120 V for 1 h. Next, the proteins were transferred to a PVDF membrane by electrophoresis at 200 mA for 2 h. The membrane was then incubated in TBS-Tween 20 (TBST) with 5% milk at room temperature for 1 h. After being rinsed with TBST, the membranes were incubated with primary antibodies (Sigma-Aldrich, CA, USA) overnight at 4 °C. The β-actin was used as a loading control for normalisation. After being rinsed in TBST three times, the membranes were incubated with a secondary antibody (Cat. no. sc-516102; 1:5000; Santa Cruz, Biotechnology, CA, USA) for 2 h at room temperature. Finally, the bands were visualised with an electrochemiluminescence substrate kit (Rahn AG) and quantified with Bio-Rad Quantity One software (Bio-Rad Laboratories, Inc.).

### Measurement of thioredoxin reductase activity

The measurement of thioredoxin reductase activity was performed with a commercial thioredoxin reductase kit (Cat. no. ab190804, Abcam, Shanghai, China) following the instruction. Briefly, cell samples were prepared as instructed and the protein concentration of extracts were determined with BCA method. Secondly, all reagents were equilibrated to room temperature. Samples were then diluted to desired protein concentration in 1X Incubation Buffer. Sample (100 µL) was added to each well to incubate for 2 h at room temperature. Next, each well was aspirated and washed three times using 300 µL 1X Wash Buffer per wash, then 200 µL Reaction Buffer was added to each well. Afterwards, the colour development was recorded at 412 nm after 30 min.

### Measurement of oxidative products and antioxidative enzymes

Briefly, Müller cells were collected and homogenised in a potassium phosphate buffer (50 mM, pH 7.5). After they were centrifuged at 1500 *g* (10 min, 4 °C), the supernatant was collected to measure the concentrations of malondialdehyde (MDA), protein carbonyl and 8-hydroxy-2-deoxyguanosine (8-OHdG). The methods were similar with previous studies (Levine et al. 2000; Weng et al. 2007; Umemura et al. 2009). The activities of the superoxide dismutase (SOD, cat. no. S0103), catalase (CAT, cat. no. S0051) and glutathione peroxidase (GPx, cat. no. S0058) were examined with corresponding assay kits purchased from Beyotime (Shanghai, China). The activity of all these enzymes was expressed as U/mg tissue.

### Detection of cellular ROS using flow cytometry

Müller cells were seeded at a density of 5 × 10^5^ cells/mL on a 6-well plate coated with polylysine. When the cells reached 80% confluence, the cells were grouped into control, vehicle, HG + 5 μM edaravone (Eda-5 μM), HG + 10 μM edaravone (Eda-10 μM), HG + 20 μM edaravone (Eda-20 μM) and HG + 40 μM edaravone (Eda-40 μM) groups. After culturing for 72 h, the cells of each group were transferred to EP tubes and washed with PBS three times. According to the instructions of the ROS detection kit, 1 mL 2′,7′-dichlorofluorescein diacetate (DCFH-DA, 10 μM) was added to each tube and mixed at 37 °C, followed by incubation in the dark for 20 min. Upon washing with serum-free medium three times, the cells were resuspended to a 500 μL cell resuspension. The fluorescence intensity of DCFH-DA was measured by flow cytometry.

### Detection of mitochondrial membrane potential by flow cytometry

Müller cells were seeded in a 6-well plate coated with polylysine (5 × 10^5^ cells/mL). When the cells reached 80% confluence, the cells were grouped into control, vehicle, HG + 5 μM edaravone (Eda-5 μM), HG + 10 μM edaravone (Eda-10 μM), HG + 20 μM edaravone (Eda-20 μM) and HG + 40 μM edaravone (Eda-40 μM) groups. After culturing for 72 h, the Müller cells were washed with PBS three times and resuspended to a 500 μL cell resuspension with culture medium. Next, 0.5 mL JC-1 staining working solution was added to the cell resuspension according to the instructions of the mitochondrial membrane potential detection kit (Beyotime, Shanghai, China). The cell resuspension was incubated for 20 min in the dark, washed twice and resuspended in JC-1 staining buffer (1×). Finally, the JC-1 signal was detected by flow cytometry.

### TRX1 overexpression and small-interfering RNA (siRNA)

For TRX1 overexpression, the method used was similar to that described in the study by Zhang et al. (2018), employing a lentiviral vector encoding TRX1. Müller cells were transfected with lentiviral vectors carrying TRX1 (TRX1^+/+^) or an empty lentiviral vector (EmptyV) using Lipofectamine 2000 transfection reagent (Invitrogen, Thermo Fisher Scientific, Inc., MA, USA). The recombinant plasmids were provided by Wuhan Transduction Bio Co, Ltd. Stably transfected cells (>80%) were then cultured in medium containing 20 mM glucose for 72 h. The expression of TRX1 was detected by western blot analysis.

For TRX1 siRNA, the method employed was similar to that reported in the study by Chen et al. (2013). Briefly, Müller cells were seeded at equal densities in 6-well plates and cultured in fresh FBS-supplemented DMEM. When the cells grew to 80% confluence, they were transfected with 50 nM TRX1 siRNA (Thermo Fisher Scientific, Inc., MA, USA) or scramble siRNA control (negative control siRNA) using DharmaFECT transfection reagent (Thermo Fisher Scientific, Inc., MA, USA) according to the manufacturer’s protocol. After 24 h, the transfection medium was removed. Next, Müller cells were washed and cultured in medium containing 20 mM glucose for 72 h. The expression of TRX1 was detected by Western blot analysis.

### Statistical analyses

Data are represented as the means ± SD. One-way analysis of variance followed by Tukey’s *post hoc* test was performed with SPSS 17.0 software (SPSS, Inc.). *p* < 0.05 was used to indicate a statistically significant difference.

## Results

### Edaravone inhibits the cell death and apoptosis caused by high glucose

**Figure 1.**
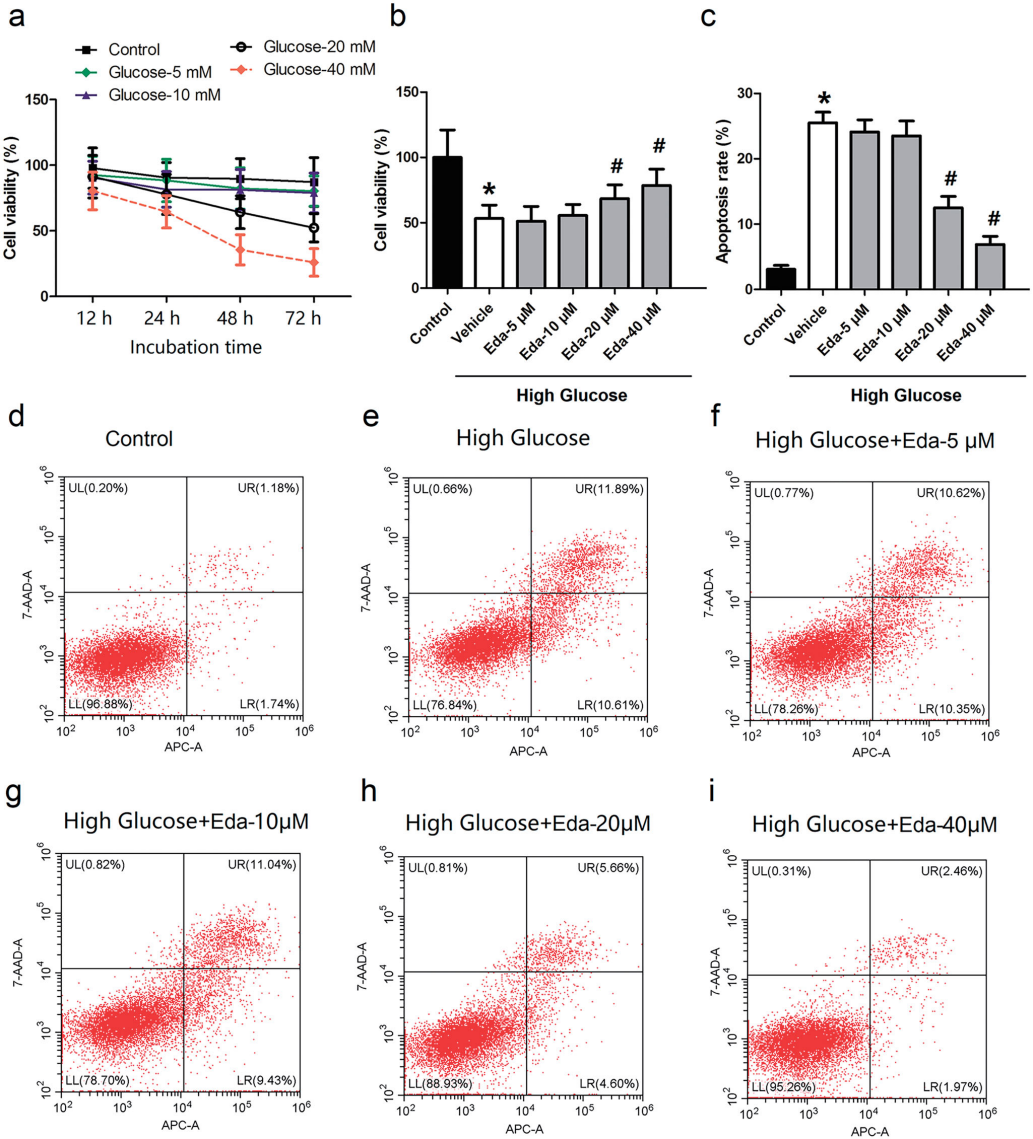
Edaravone inhibits the cell death and apoptosis caused by high glucose. Figure 1(a) shows the cell viability of Müller cells after they were treated with different concentrations of glucose for different durations. Figure 1(b) shows the effect of different concentrations of edaravone on cell viability. Figure 1(c) shows the effect of different concentrations of edaravone on cell apoptosis. Figure 1(d-i) shows the representative images of apoptosis measured by flow cytometry. Eda: edaravone. *p < 0.05 compared to the control group. ^#^*p* < 0.05 compared to the vehicle group. *N* = 12.

Müller cells were treated with glucose (5, 10, 20, and 40 mM) for different durations (12, 24, 48 and 72 h), and the cell viability was then measured by the CCK-8 method. The IC_50_ of glucose at 12, 24, 48, 72 h is 489.3, 103.5, 27.92 and 20.71 mM, respectively. As shown in Figure 1(a), when Müller cells were cultured in 20 mM glucose for 72 h, the cell viability was ∼50%. Therefore, this incubation concentration and duration was selected for the following experiments. Figure 1(b) shows the effect of different concentrations of edaravone on cell viability. It was observed that edaravone (20 and 40 μM) significantly increased cell viability compared with that of the vehicle (*p* < 0.05). The EC_50_ of edaravone is 34.38 μM. Figure 1(c) shows the effect of different concentrations of edaravone on cell apoptosis. It was observed that edaravone (20 and 40 μM) significantly decreased cell viability compared with that of the vehicle (*p* < 0.05). Figure 1(c–i) contain representative images of apoptosis, as measured by flow cytometry.

### Edaravone alters the expression of apoptotic proteins

**Figure 2.**
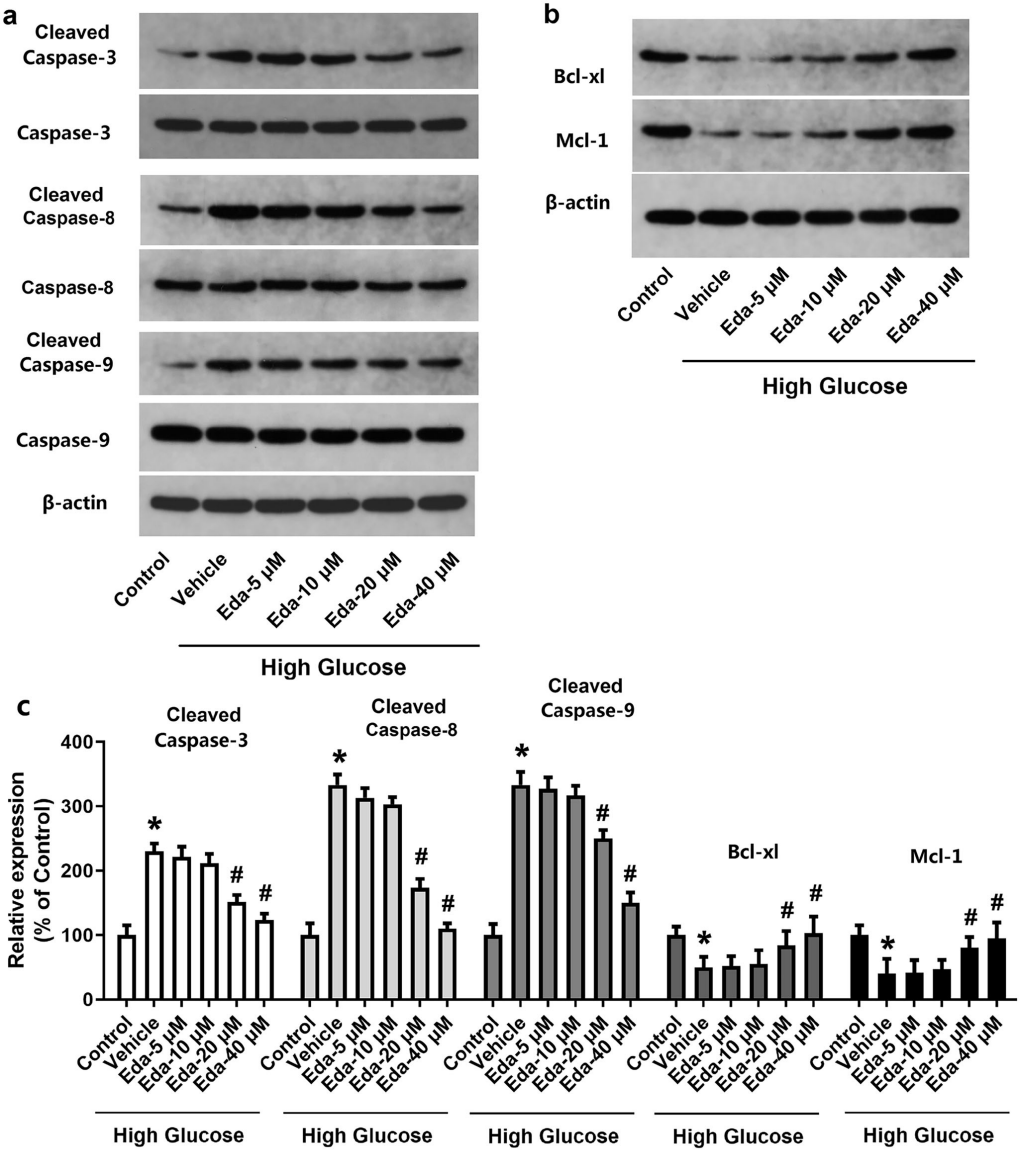
Edaravone changes the expression of apoptotic proteins. Figure 2(a and b) show the representative images of western blot bands of apoptotic proteins (cleaved caspase-3, caspase-3, cleavedcaspase-8, caspase-8, cleaved caspase-9, caspase-9, Bcl-xl and Mcl-1). Figure 2(c) shows the fold-change in the expression of apoptotic proteins. Eda: edaravone. *p < 0.05 compared to the control group. ^#^*p* < 0.05 compared to the vehicle group. *N* = 12.

Figure 2(a,b) contain representative images of western blot bands of apoptotic proteins (cleaved caspase-3, caspase-3, cleaved caspase-8, caspase-8, cleaved caspase-9, caspase-9, Bcl-xl, and Mcl-1). Figure 2(c) shows the fold-change in the ratios of cleaved caspase-3/caspase-3, cleaved caspase-8/caspase-8, cleaved caspase-9/caspase-9 and the expression of apoptotic proteins (Bcl-xl and Mcl-1) compared with that of the Control. As shown in Figure 2(c), high glucose caused a significant increase in the ratios of cleaved caspase-3/caspase-3, cleaved caspase-8/caspase-8 and cleaved caspase-9/caspase-9 and a significant decrease in Bcl-xl and Mcl-1. It was noticed that edaravone (20 and 40 μM) significantly decreased the ratios of cleaved caspase-3/caspase-3, cleaved caspase-8/caspase-8 (*p* < 0.05), but significantly increased the expression of Bcl-xl and Mcl-1 (*p* < 0.05). These results indicated that edaravone inhibited apoptosis in Müller cells cultured in high glucose.

### Edaravone inhibits oxidative stress in Müller cells cultured in high glucose

**Figure 3.**
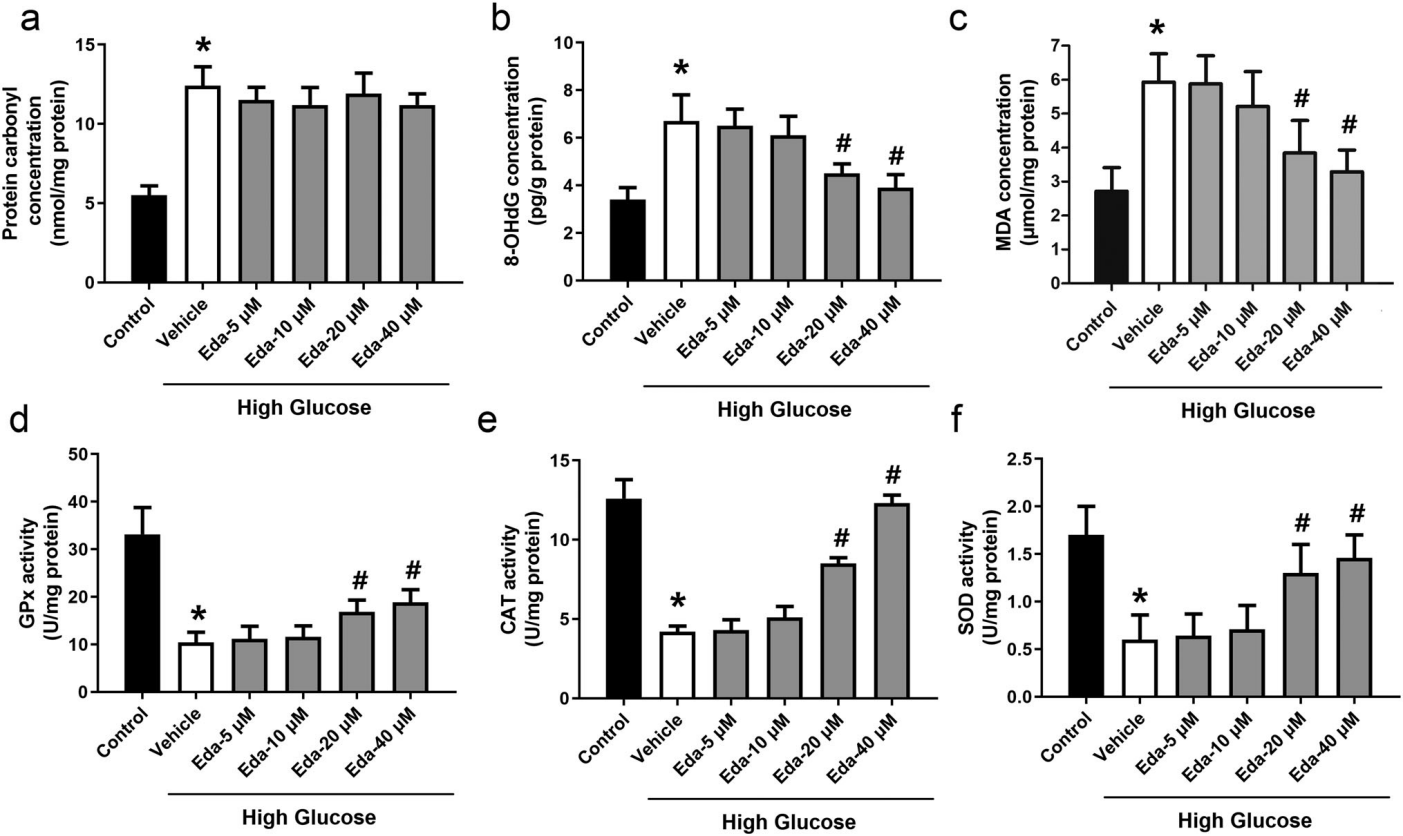
Edaravone inhibits oxidative stress in Müller cells cultured in high glucose. Figure 2(a–c) shows the changes in oxidative injury indicators (protein carbonyl, 8-OHdG and MDA). Figure 3(d–f) shows the changes in antioxidative enzyme activities (GPx, CAT and SOD). Eda: edaravone. 8-OHdG: 8-hydroxy-desoxyguanosine; MDA: malondialdehyde; GPx: glutathione peroxidase; CAT: catalase; SOD: superoxide dismutase. *p < 0.05 compared to the control group. ^#^*p* < 0.05 compared to the vehicle group. *N* = 12.

Figure 2(a–c) shows the changes in oxidative injury indicators (protein carbonyl, 8-OHdG and MDA). High glucose caused significant increases in protein carbonyl, 8-OHdG and MDA compared with those of the control. Edaravone did not cause a significant change in the level of protein carbonyl, but edaravone (20 and 40 μM) significantly decreased the levels of 8-OHdG and MDA (*p* < 0.05). Figure 3(D–F) shows the changes in antioxidative enzyme activities (GPx, CAT and SOD). Edaravone at 20 and 40 μM significantly increased the activities of GPx, CAT and SOD compared with those of the vehicle (*p* < 0.05).

### Edaravone reduces ROS levels and restores the mitochondrial membrane potential in Müller cells cultured in high glucose

**Figure 4.**
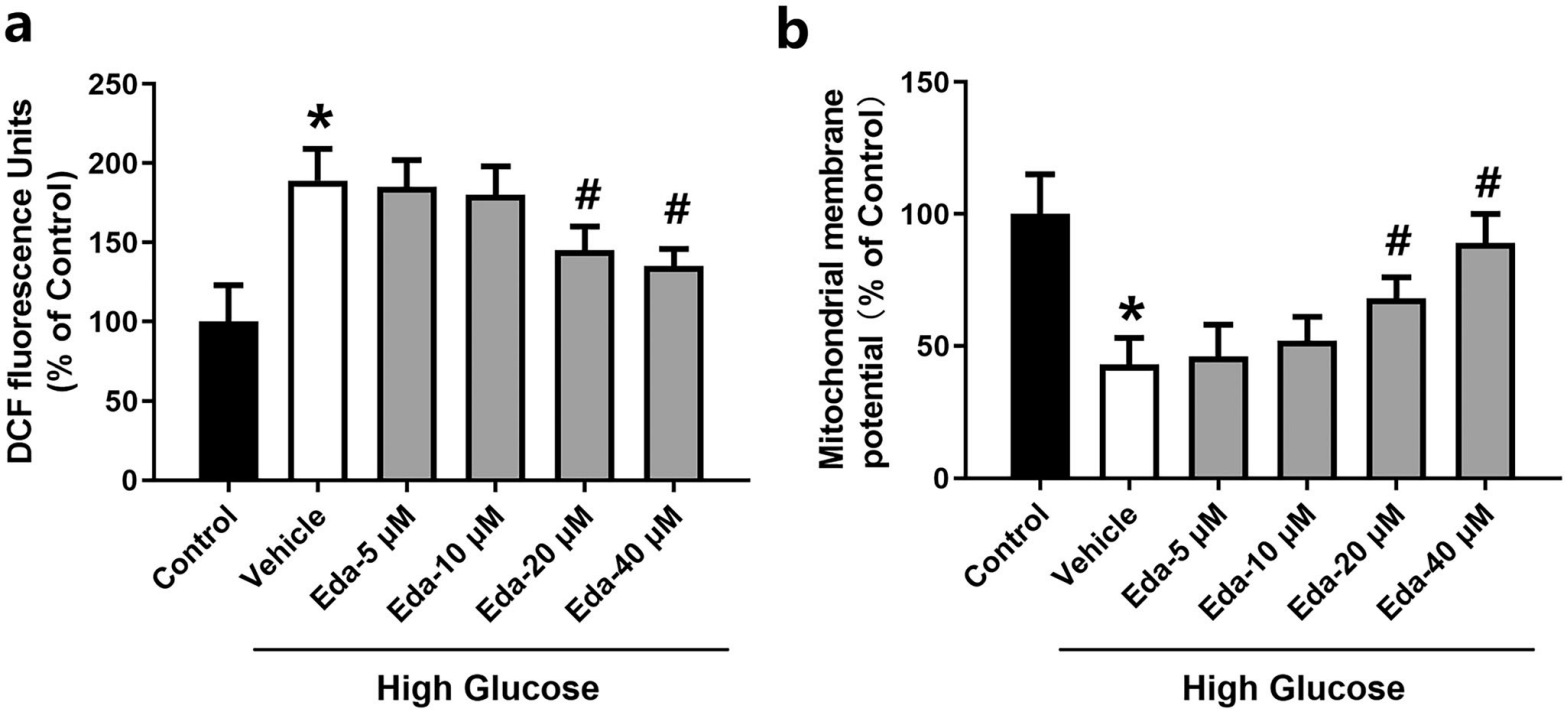
Edaravone reduces ROS levels and restores the mitochondrial membrane potential in Müller cells cultured in high glucose. Figure 4(a) shows the changes in DCF fluorescence signal. Figure 4(b) shows the changes in mitochondrial membrane potential. Eda: edaravone. DCF: 2′,7′-dichlorodihydrofluorescein. ROS: reactive oxygen species. *p < 0.05 compared to the control group. ^#^*p* < 0.05 compared to the vehicle group. *N* = 12.

Figure 4(a) shows the changes in DCF fluorescence signal, which is a ROS indicator, in Müller cells. High glucose caused a significant increase in DCF fluorescence compared with that of the control (*p* < 0.05). Edaravone (20 and 40 μM) significantly decreased DCF fluorescence compared with that of the vehicle (*p* < 0.05). Figure 4(b) shows the changes in mitochondrial membrane potential in Müller cells. High glucose caused a significant decrease in the mitochondrial membrane potential compared with that of the control (*p* < 0.05). Edaravone (20 and 40 μM) significantly increased the mitochondrial membrane potential compared with that of the vehicle (*p* < 0.05).

### Edaravone alters the expression of TRX1, PGC-1α, NRF1 and TFAM and the thioredoxin reductase activity

**Figure 5.**
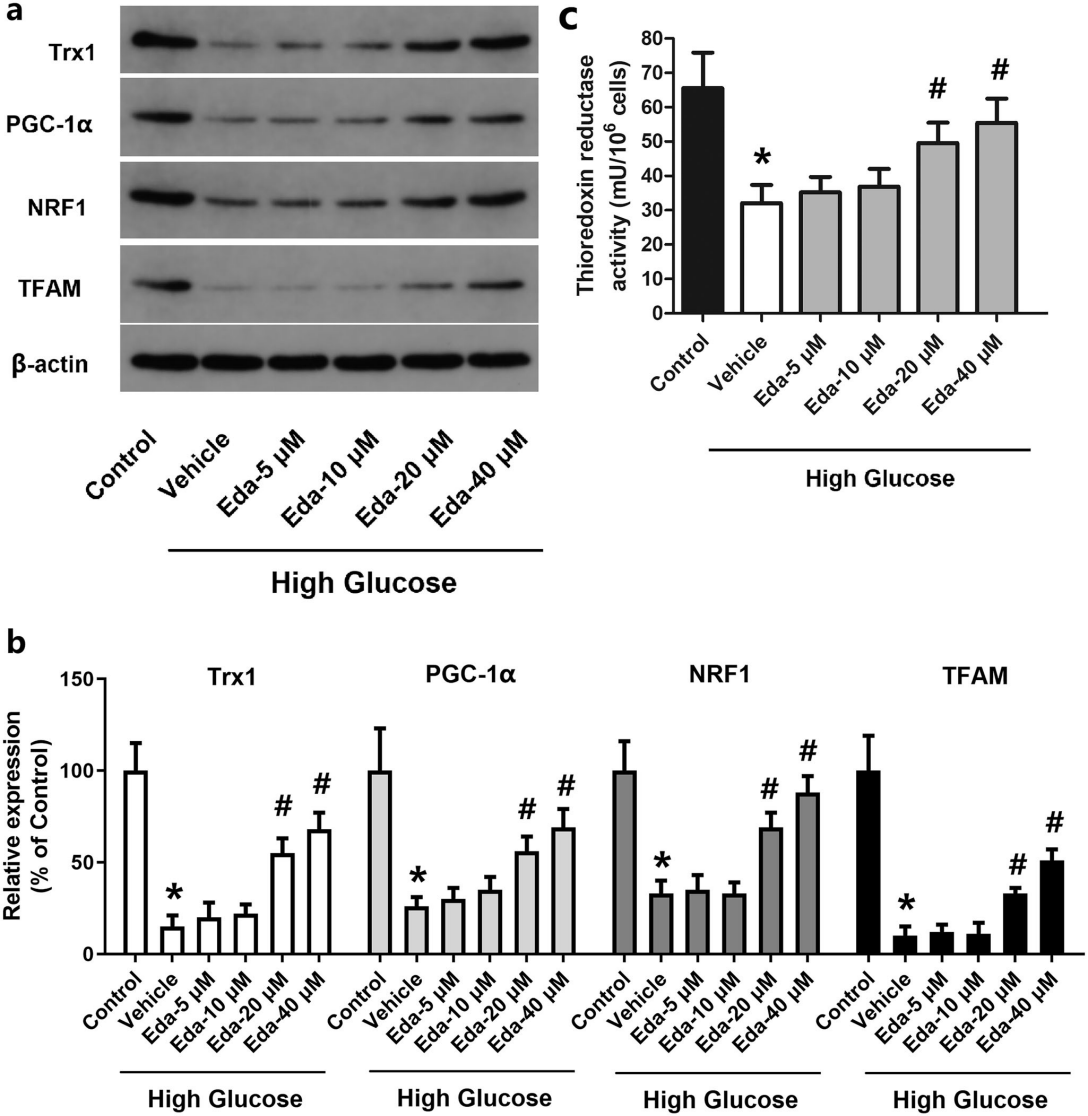
Edaravone alters the expression of TRX1, PGC-1α, NRF1 and TFAM and thioredoxin reductase activity. Figure 5(a) shows representative images of western blots of TRX1, PGC-1α, NRF1 and TFAM; Figure 5(b) shows the fold-change in the expression of these proteins compared with that of the control; Figure 5(c) shows the changes of thioredoxin reductase activity. TRX1: thioredoxin1; PGC-1α: peroxisome proliferator-activated receptorγcoactivator-1α; NRF1: Nuclear respiratory factor 1; TFAM: mitochondrial transcription factor A; Eda: edaravone. *p < 0.05 compared to the control group. ^#^*p* < 0.05 compared to the vehicle group. *N* = 12.

Figure 5(a) shows representative images of western blots of TRX1, PGC-1α, NRF1 and TFAM, while Figure 5(b) shows the fold-change in the expression of these proteins compared with that of the control. It was shown that high glucose caused a significant decrease in TRX1, PGC-1α, NRF1 and TFAM compared with that of the control (*p* < 0.05). By contrast, edaravone (20 and 40 μM) significantly increased the expression of TRX1, PGC-1α, NRF1 and TFAM compared with that of the vehicle (*p* < 0.05). As shown in Figure 5(c), high glucose caused a significant decrease in the thioredoxin reductase activity, which was significantly increased by edaravone (20 and 40 μM).

### Effect of TRX1 overexpression or siRNA on cell viability, apoptosis, ROS levels and mitochondrial membrane potential in Müller cells

**Figure 6.**
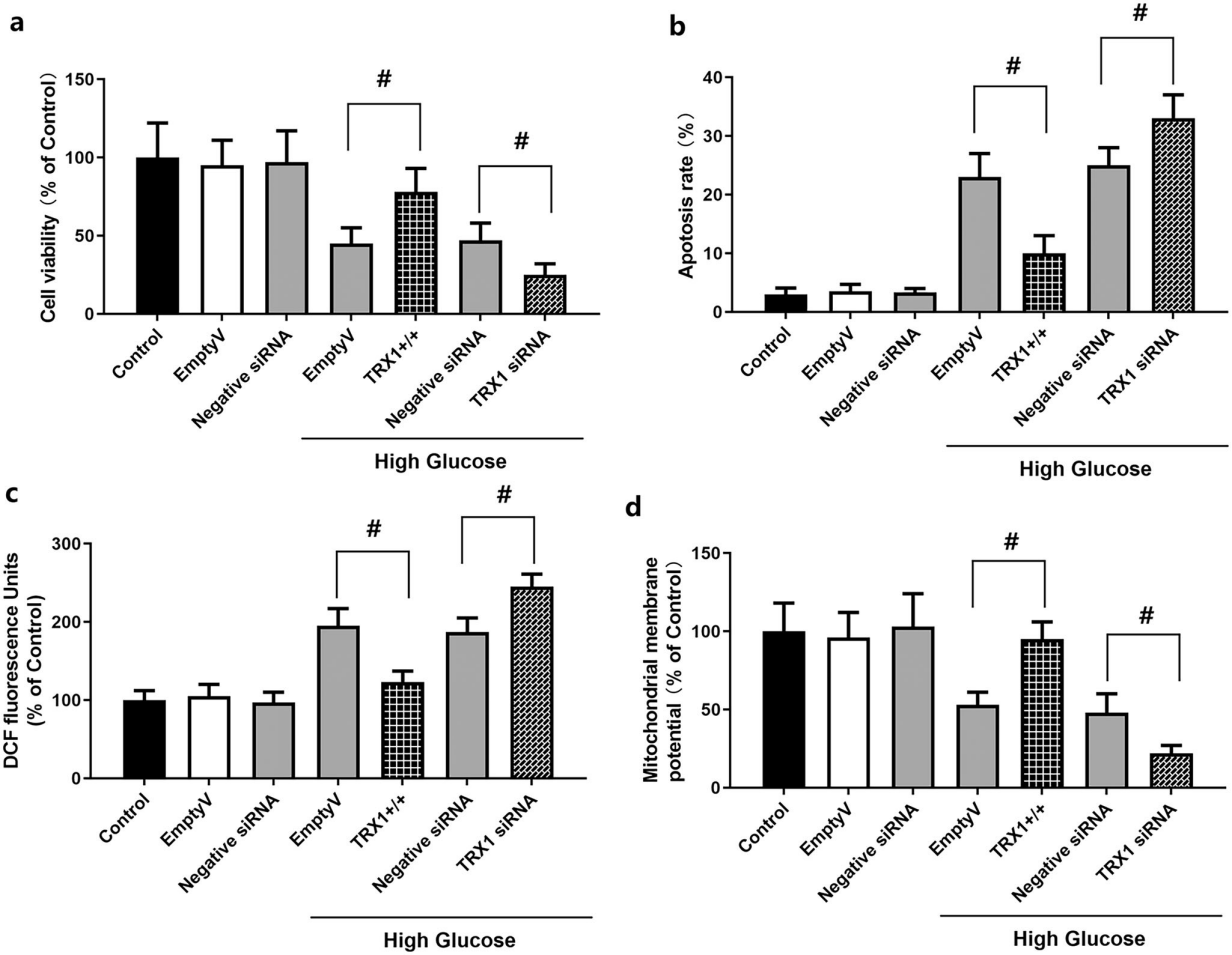
Effect of TRX1 overexpression or siRNA on cell viability, apoptosis, ROS levels and mitochondrial membrane potential in Müller cells. Figure 6(a) shows the effect of TRX1 overexpression or siRNA on cell viability. Figure 6(b) shows the effect of TRX1 overexpression or siRNA on cell apoptosis. Figure 6(c) shows the effect of TRX1 overexpression or siRNA on ROS levels. Figure 6(d) shows the effect of TRX1 overexpression or siRNA on mitochondrial membrane potential. TRX1: thioredoxin1; ROS: reactive oxygen species. ^#^*p* < 0.05 between groups. *N* = 12.

As shown in Figure 6, empty lentiviral vector or scramble siRNA control (negative control siRNA) did not induce significant changes in cell viability, apoptosis, ROS levels or mitochondrial membrane potential in Müller cells compared with those observed in the control. Figure 6(a) shows the effect of TRX1 overexpression or siRNA on cell viability. When cells were cultured in high glucose, TRX1 overexpression significantly increased the cell viability compared with that of the empty lentiviral vector (*p* < 0.05), while TRX1 siRNA significantly decreased the cell viability compared with that of the negative control siRNA (*p* < 0.05). Figure 6(b) shows the effect of TRX1 overexpression or siRNA on cell apoptosis. TRX1 overexpression significantly inhibited cell apoptosis compared with that of the empty lentiviral vector (*p* < 0.05), while TRX1 siRNA significantly increased cell apoptosis compared with that of negative control siRNA (*p* < 0.05). Figure 6(c) shows the effect of TRX1 overexpression or siRNA on ROS levels. TRX1 overexpression significantly decreased ROS levels compared with those of the empty lentiviral vector (*p* < 0.05), while TRX1 siRNA significantly increased ROS levels compared with that of the negative control siRNA (*p* < 0.05). Figure 6(d) shows the effect of TRX1 overexpression or siRNA on mitochondrial membrane potential. TRX1 overexpression significantly increased the mitochondrial membrane potential compared with that of the empty lentiviral vector (*p* < 0.05), while TRX1 siRNA significantly decreased the mitochondrial membrane potential compared with that of the negative control siRNA (*p* < 0.05).

### Effect of TRX1 overexpression or siRNA on the expression of TRX1, PGC-1α, NRF1 and TFAM and the thioredoxin reductase activity

**Figure 7.**
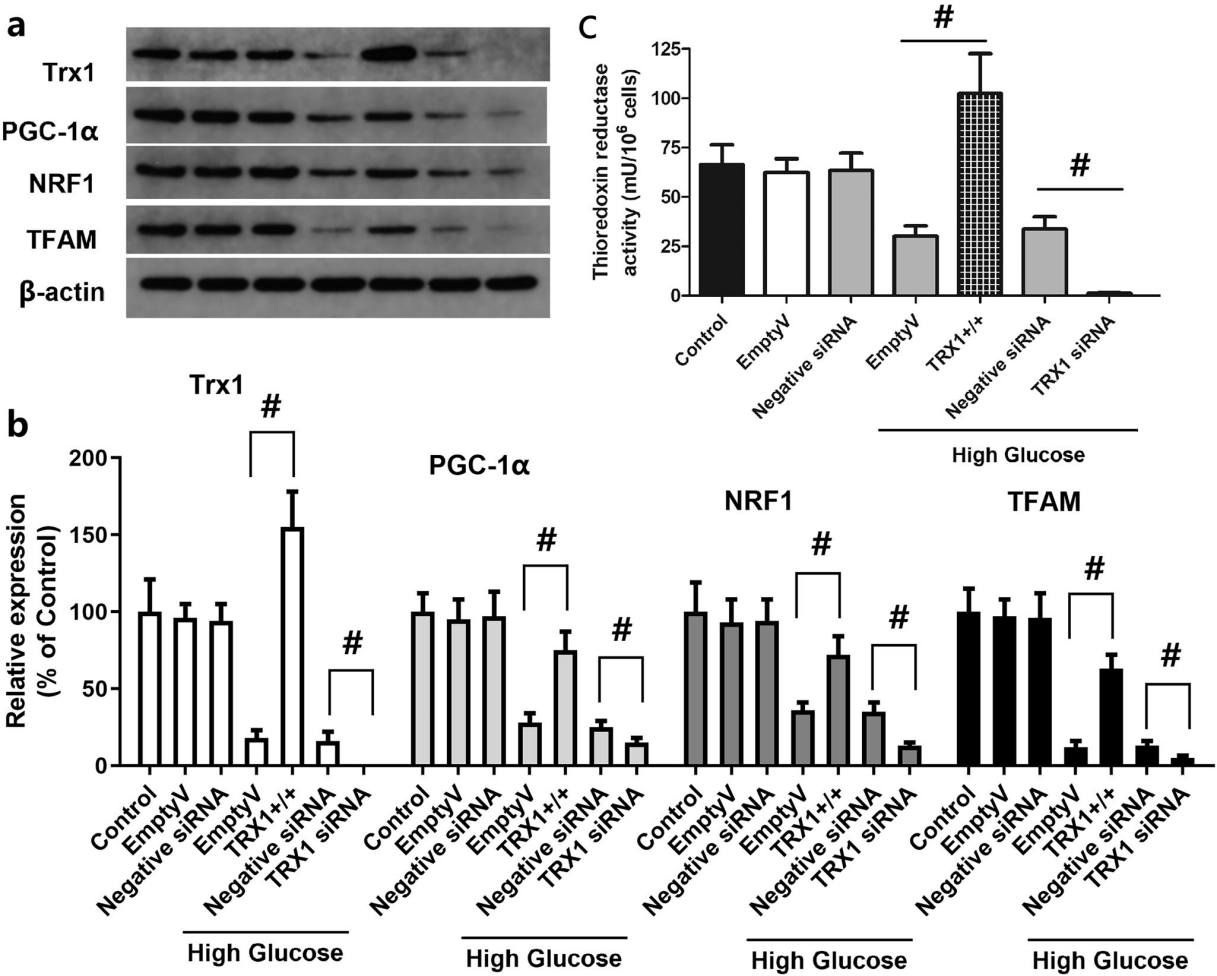
Effect of TRX1 overexpression or siRNA on the expression of TRX1, PGC-1α, NRF1 and TFAM and thioredoxin reductase activity. Figure 7(a) shows representative western blot images of TRX1, PGC-1α, NRF1 and TFAM; Figure 7(b) shows the fold-change in the expression of these proteins compared with that of the control; Figure 7(c) shows the changes of thioredoxin reductase activity. TRX1 overexpression significantly increased the expression of TRX1, PGC-1α, NRF1 and TFAM and thioredoxin reductase activity; TRX1 siRNA significantly decreased their expression and the thioredoxin reductase activity compared with that of the negative control siRNA. TRX1: thioredoxin1; PGC-1α: peroxisome proliferator-activated receptorγcoactivator-1α; NRF1: Nuclear respiratory factor 1; TFAM: mitochondrial transcription factor A; Eda: edaravone. ^#^*p* < 0.05 between groups. *N* = 12.

Figure 7(a) shows representative western blot images of TRX1, PGC-1α, NRF1 and TFAM, while Figure 7(b) shows the fold-changes in the expression of these proteins compared with that of the control. Empty lentiviral vector or scramble siRNA control (negative control siRNA) did not induce significant change in the expression of TRX1, PGC-1α, NRF1 or TFAM, whereas TRX1 overexpression significantly increased the expression of TRX1, PGC-1α, NRF1 and TFAM compared with that of the empty lentiviral vector (*p* < 0.05), and TRX1 siRNA significantly decreased their expression compared with that of the negative control siRNA (*p* < 0.05). As shown in Figure 7(c), empty lentiviral vector or scramble siRNA control (negative control siRNA) did not significantly change the thioredoxin reductase activity, but TRX1 overexpression significantly increased it. TRX1 siRNA significantly decreased the thioredoxin reductase activity compared with the negative control siRNA (*p* < 0.05).

## Discussion

Retinopathy is one of the most common and serious microvascular complications of diabetes in the eye. Its incidence and severity increase with the prolongation of the course of diabetes. If the course of diabetes is ≤5 years, the incidence of DR can reach 44.4%, and if the course of diabetes is up to 7 years, the incidence of retinopathy can be >56% (Malone et al. 2001). High glucose status is the basis for the occurrence and development of DR. The occurrence of DR is closely associated with the increase in blood sugar (Antonetti et al. 2012; Chavira-Suárez et al. 2012). The organelles in retinal Müller cells may be damaged under long-term hyperglycaemia, and changes in cell ultrastructure can stimulate Müller cell activation and cause dysfunction (Abu El-Asrar et al. 2001; Wang et al. 2010). The deterioration of Müller and nerve cells often occurs before retinopathy microangiopathy (Mizutani et al. 1998; Bringmann et al. 2006; Le 2017). The neurons and/or glial cells of the retina may be particularly sensitive to the effects of long-term hyperglycaemia, and microvascular damage becomes the secondary to its metabolic disorders (Lieth et al. 1998). In the present study, Müller cells were treated with different concentrations of glucose (5, 10, 20, and 40 mM) for different durations (12, 24, 48 and 72 h), and then the cell viability was measured. The results showed that the cell viability was significantly decreased with the increase in the concentration of glucose or the incubation time. Treatment of Müller cells with 20 mM glucose for 72 h induced significant apoptosis and increased the expression of apoptotic proteins (caspase-3, caspase-8 andcaspase-9). These results clearly demonstrated that high glucose treatment could induce Müller cell death and apoptosis.

ROS can not only directly cause damage to proteins, lipids, carbohydrates and DNA, but can also change gene expression by stimulating signalling pathways, thus causing cell damage (Ha and Lee 2000). ROS are also involved in the regulation of the expression of pro-fibrosis genes caused by hyperglycaemia and in the occurrence of proliferative DR (Lee et al. 2003). Increased ROS levels lead to enhanced oxidative stress, which is considered to be the initiating step of various microvascular complications of diabetes (Ha and Lee 2000). The results of the present study suggest that high glucose caused significant increases in protein carbonyl, 8-OHdG and MDA, and impaired the activities of antioxidative enzymes (GPx, CAT and SOD) in Müller cells. It was shown that the ROS level in Müller cells was directly increased by high glucose treatment, which was consistent with previous studies (Rösen et al. 2001; Brownlee 2005; Rolo and Palmeira 2006). To investigate the effect of edaravone, an antioxidative drug on hyperglycaemia-induced cell injury, Müller cells were treated with high glucose and different concentrations of edaravone, and then cell viability, cell apoptosis and oxidative stress were measured in Müller cells. The results showed that higher concentrations of edaravone (20 and 40 μM) significantly increased cells from the cell death, apoptosis and oxidative stress induced by high glucose, suggesting that antioxidative treatments could be effective for preventing hyperglycaemia-induced cell injury.

Mitochondria, as the main organelle for cells to perform biological oxidation and energy conversion, is the main source of ROS and the main target of oxidative injury (Li et al. 2008). When mitochondrial dysfunction occurs, the mitochondrial membrane potential decreased and the mitochondrial membrane channel opens, which is a key step to initiate cell apoptosis (Ly et al. 2003). ROS can cause a decrease in mitochondrial membrane potential and leakage of cytochrome C, and then induce cell apoptosis by activating the downstream cascade reaction (Anuradha et al. 2001). As shown in Figure 4, high glucose caused significant decrease in the mitochondrial membrane potential. The mitochondrial membrane potential in the high glucose group was ∼50% of that of the control. Co-treatment with low concentrations of edaravone (5 and 10 μM) did not cause a significant change in the mitochondrial membrane potential, while higher concentrations of edaravone (20 and 40 μM) significantly increased it. These results indicated that the impact of edaravone on mitochondrial membrane potential may be an important mechanism of its protection against high glucose-induced cell injury. The release of cytochrome *c* can activate caspase-3 and cause mitochondrial respiration chain inhibition, loss of membrane potential and termination of glycolysis, eventually leading to cell death. Similarly, Kowluru and Koppolu (2002) reported that under enhanced cell oxidative stress, apoptosis can be caused by the activation of the caspase-3 apoptotic pathway, while antioxidants could decrease apoptosis by inhibiting the caspase-3 pathway.

To investigate the possible involvement of TRX and the PGC-1α/NRF1/TFAM pathway, the expression of TRX1, PGC-1α, NRF1 and TFAM and thioredoxin reductase activity was measured following treatment of Müller cells with high glucose and edaravone. The expression of these molecules and thioredoxin reductase activity was significantly decreased by high glucose, but partly recovered by treatment with edaravone (20 and 40 μM). Previous studies revealed the impact of high glucose on the expression of TRX1, PGC-1α, NRF1 and TFAM. In the study by Luan et al. (2009), the activity of TRX1 was decreased in diabetic cardiac tissues. As suggested by Hou et al. (2020), the mechanism may involve TRX-interacting protein (TXNIP), an endogenous inhibitor of TRX. It was overexpressed in diabetic myocardium due to the carbohydrate response element within its promoter. Hyperglycaemia causes overexpression of TXNIP, which interacts with TRX1 and inhibits its nuclear translocation, thus blocking TRX1-dependent gene transcription and the expression of genes associated with cell death and survival (Dunn et al. 2010). Zhang et al. (2018) reported that high glucose caused a time-dependent decrease in the expression of PGC-1α, NRF1 and TFAM in mouse podocytes, and an increase in ROS levels in cells and mitochondria. The study by Nanjaiah and Vallikannan (2019) revealed that hyperglycaemia affected mitochondria in the retina, and diminished mitochondrial biogenesis by downregulation of PGC-1α and TFAM. To further explore the mechanism of action of TRX1, TRX1 overexpression and siRNA methods were used to examine the changes in cell viability, apoptosis, ROS generation and mitochondrial potential. The results showed that TRX1 overexpression significantly increased cell viability, inhibited cell apoptosis, decreased ROS levels and significantly increased the mitochondrial potential, while TRX1 siRNA exerted the opposite effects. Furthermore, TRX1 overexpression significantly increased the expression of TRX1, PGC-1α, NRF1 and TFAM and thioredoxin reductase activity, while TRX1 siRNA significantly decreased the expression of these molecules and thioredoxin reductase activity, indicating the regulatory role of TRX1 on the PGC-1α/NRF1/TFAM pathway. The study by Ago et al. *(*2006) also indicated that, in cardiac myocytes, TRX1 upregulates mitochondrial proteins and enhances mitochondrial functions via PGC-1α and NRFs. The expression of PGC-1α and NRF1 was upregulated by TRX1 overexpression, which promoted the transcriptional activity of NRF1 and NRF2 (Ago et al. 2006). These results suggested that TRX1 may be a key molecule in the effects of edaravone against high glucose-induced cell injury.

## Conclusions

Our study revealed that high glucose increased oxidative stress in retinal Müller cells, causing cell damage and even apoptosis, while edaravone could be used as an antioxidant to reduce ROS levels and enhance the antioxidant capacity of cells. The limitation of the present study is that these results only proved that the effect of edaravone is anti-oxidative stress, not specifically to diabetes. Furthermore, the underlying relationship between TRX1 and PGC-1α/NRF1/TFAM pathway needs further exploration. The main novelty is that edaravone could restore the expression of TRX1, which activated the PGC-1α/NRF1/TFAM pathway to stabilise mitochondrial membrane structure and improve mitochondrial function, thereby inhibiting the cell apoptosis caused by oxidative stress under high-glucose conditions. This study suggested that TRX1 and the PGC-1α/NRF1/TFAM pathway may be explored as new molecular targets for DR. These findings provide us a new potential approach (edaravone or other antioxidant) for the treatment of DR. New medicines that target TRX1 and the PGC-1α/NRF1/TFAM pathway may be developed to treat DR.

## Data Availability

The datasets used and/or analysed during the current study are available from the corresponding author on reasonable request.
